# A BRCA1 deficient, NFκB driven immune signal predicts good outcome in triple negative breast cancer

**DOI:** 10.18632/oncotarget.7865

**Published:** 2016-03-02

**Authors:** Niamh E. Buckley, Paula Haddock, Ricardo De Matos Simoes, Eileen Parkes, Gareth Irwin, Frank Emmert-Streib, Stephen McQuaid, Richard Kennedy, Paul Mullan

**Affiliations:** ^1^ Centre for Cancer Research and Cell Biology, Queen's University Belfast, Belfast, UK

**Keywords:** triple negative breast cancer, BRCA1, NFkB, predictive biomarker, microenvironment

## Abstract

Triple negative (TNBCs) and the closely related Basal-like (BLBCs) breast cancers are a loosely defined collection of cancers with poor clinical outcomes. Both show strong similarities with BRCA1-mutant breast cancers and BRCA1 dysfunction, or ‘BRCAness’, is observed in a large proportion of sporadic BLBCs. BRCA1 expression and function has been shown *in vitro* to modulate responses to radiation and chemotherapy. Exploitation of this knowledge in the treatment of BRCA1-mutant patients has had varying degrees of success. This reflects the significant problem of accurately detecting those patients with BRCA1 dysfunction. Moreover, not all BRCA1 mutations/loss of function result in the same histology/pathology or indeed have similar effects in modulating therapeutic responses. Given the poor clinical outcomes and lack of targeted therapy for these subtypes, a better understanding of the biology underlying these diseases is required in order to develop novel therapeutic strategies.

We have discovered a consistent NFκB hyperactivity associated with BRCA1 dysfunction as a consequence of increased Reactive Oxygen Species (ROS). This biology is found in a subset of BRCA1-mutant and triple negative breast cancer cases and confers good outcome. The increased NFκB signalling results in an anti-tumour microenvironment which may allow CD8+ cytotoxic T cells to suppress tumour progression. However, tumours lacking this NFκB-driven biology have a more tumour-promoting environment and so are associated with poorer prognosis. Tumour-derived gene expression data and cell line models imply that these tumours may benefit from alternative treatment strategies such as reprogramming the microenvironment and targeting the IGF and AR signalling pathways.

## INTRODUCTION

Breast cancer is a heterogeneous disease comprising of multiple tumour types that require different treatment approaches and have varied patient outcomes. Patient stratification, based on expression of the estrogen (ERα) or Her2/neu/ERBB2 (Her2) receptors, has allowed for the use of targeted therapies such as Tamoxifen and Trastuzamab, respectively. Breast cancers that do not express these receptors are termed “triple negative breast cancers” (TNBCs) and have the poorest clinical outcome, reflecting the fact that they lack targeted therapies. All TNBCs are currently treated with DNA-damaging chemotherapy regimes such as FEC (5-FU, Epirubucin and Cyclophosphamide). They are a poorly defined subgroup with a large degree of heterogeneity suggesting that optimal treatment may only be attained by use of different treatment regimens. Gene expression microarray analyses of tumours has allowed breast cancers to be re-classified. These include the ERα and Her2 positive subgroups in addition to a Basal-like (BLBC) subgroup which is associated with the poorest clinical outcomes [1]. There is a high degree of overlap between the TNBC and BLBC subgroups with up to 70% of TNBCs displaying BLBC gene profiles as well as 77% of BLBCs classified as TNBC [2].

Hereditary breast cancers arising from mutation of the tumour suppressor gene, BRCA1, closely resemble sporadic TNBC/BLBCs while BRCA1 expression is downregulated in up to 30% of sporadic BLBCs [3]. BRCA1 function can therefore be abrogated both by mutation and downregulation of expression (‘BRCAness’) leading to tumourigenesis [4]. BRCA1 expression and function has been shown *in vitro* to modulate responses to radiation and chemotherapy[5, 6]. Exploitation of this knowledge in the treatment of BRCA1-mutant patients has had varying degrees of success [7, 8]. This reflects the significant problem of accurately detecting those patients with BRCA1 dysfunction. Moreover, not all BRCA1 mutations/loss of function result in the same histology/pathology or indeed have similar effects in modulating therapeutic responses [9, 10]. Therefore, an increased understanding of how discrete modes of BRCA1 dysfunction modulate disease biology and therapeutic responses is required.

BRCA1 tumours often display high numbers of infiltrating lymphocytes both intratumoural and in the surrounding stroma [11]. Moderate to extensive lymphocytic infiltrate (LI) is also observed in about half of all TNBC cases [12] and this is associated with good clinical outcome [12-14]. Indeed, a number of gene signatures based on activation of immune signalling have been developed in TNBCs, which predict for good outcome to current standard of care chemotherapy [15-19]. Furthermore, it has been shown that BRCA1 mutant tumours tend to overexpress immune response genes [17, 20]. Whilst these studies suggest a role for the immune system in modulating TNBC responses, they do not elucidate the biology underlying the up-regulation of the immune response genes and the functional significance of the genes themselves in tumourigenesis.

The NFκB pathway primarily mediates the cellular response to external stimuli and plays a crucial role in regulating the immune response. Activation of the NFκB pathway underpins many aspects of cancer including survival, invasion and metastasis. BRCA1 has been shown to interact with p65 and acts as a transcriptional co-activator in response stimuli [21] and NFκB acts a critical mediator of BRCA1-induced chemoresistance [22]. However, in this study, we demonstrate that in the absence of functional BRCA1, basal NFκB activity is increased and NFκB target genes are increased in TNBC cell lines. A BRCA1 deficient, NFκB driven immune signal has been identified and this predicts good clinical outcome in TNBCs. This is underpinned by a favourable “M1-type” macrophage tumour microenvironment promoting active cytotoxic CD8+ infiltrate.

## RESULTS

**Figure 1:**
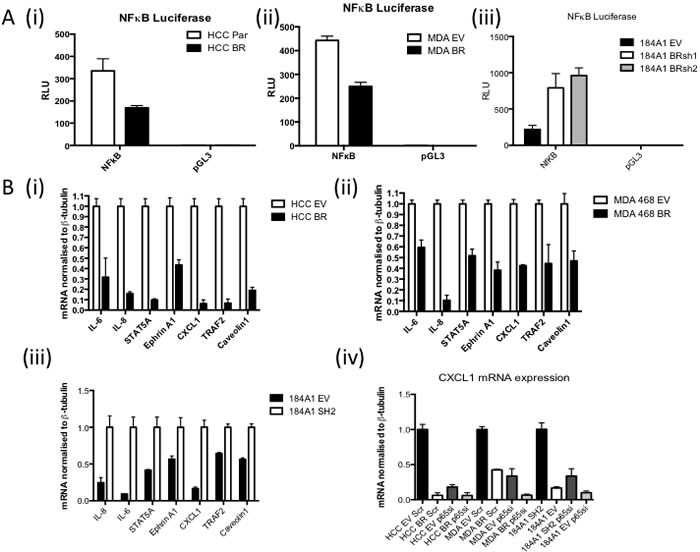
**A.** NFκB Luciferase Activity Assay of (i) HCC1937 (BRCA1 mutant) cells stably transfected with either empty vector (HCC EV) or full length BRCA1 (HCC BR), (ii) MDA468 (BRCA1 low) cells stably transfected with either empty vector (MDA EV) or full length BRCA1 (MDA BR), or (iii) 184A1 (normal breast) cells stably transfected with empty vector (EV) or BRCA1 shRNA (BRsh2). Cells were transfected with either NFkB reporter construct (NFκB) or the empty vector control (pGL3). Renilla was used to normalise for transfection efficiency. Values are expressed as relative luciferase units (RLU) normalised to pGL3 and Renilla. **B.** Real time PCR of NFκB target genes in (i) HCC EV and BR, (ii) MDA EV and BR and (iii) 184A1 EV and SH2 cells. β-tubulin was used as a housekeeper. Expression was then normalised to HCC EV, MDA EV and 184A1 SH2 respecively (iv) Real time PCR analysis of CXCL1 mRNA in HCC EV and BR, MDA EV and BR and 184A1 EV and SH2 cells transiently transfected with either scrambled control (scr) or p65 specific (p65si) siRNA for 72hrs. β-tubulin was used as a housekeeper.

Using a NFκB luciferase reporter assay, higher NFκB activity is observed in BRCA1 mutant HCC1937 (Figure 1A(i)) and BRCA1 low MDA-MB-468 (Figure 1A(ii)) cells compared to their isogenic matched BRCA1 reconstituted controls. Conversely, shRNA mediated BRCA1 knockdown in the 184A1 normal breast cell line results in increased NFκB activity ((Figure 1A(iii)). Increased expression of known NFκB target genes was also observed in the absence of functional BRCA1 expression (Figure 1B(i-iii)). SiRNA against the p65 subunit of NFκB was used to demonstrate the increased expression in the absence of functional BRCA1 is dependent on the increased NFκB activity using CXCL1 as an exemplar NFkB-dependent gene (Figure 1B(iv)).

**Table 1:** Univariate Cox Proportional Hazard Ratio analysis of survival in the in-house TNBC and the publically available GSE58812, GSE21653 and GSE2034 data sets

Relapse Free Survival			Cox PH - Univariate		
			HR	%95 CI	*p*-value
Inhouse TNBC		N(n) 60 (19)			
BRCA1/NFkB	off	37 (15)	1		
	on	23 (4)	0.3559	0.118-1.073	0.0666
GSE58812		N(n) 107(31)			
BRCA1/NFkB	off	90 (31)	1		
	on	17 (0)	0.2886 *	0.1179-0.7065	0.0065
GSE21653		N(n) 85 (27)			
BRCA1/NFkB	off	62 (25)	1		
	on	23 (2)	0.1956	0.04632-0.8263	0.0264
GSE2034		N(n) 77 (27)			
BRCA1/NFkB	off	42 (19)	1		
	on	35 (8)	0.4412	0.1929-1.009	0.0525

* Cox PH HR not possible as no events in NFkB on, Mantel-Haenszel HR shown

**Figure 2:**
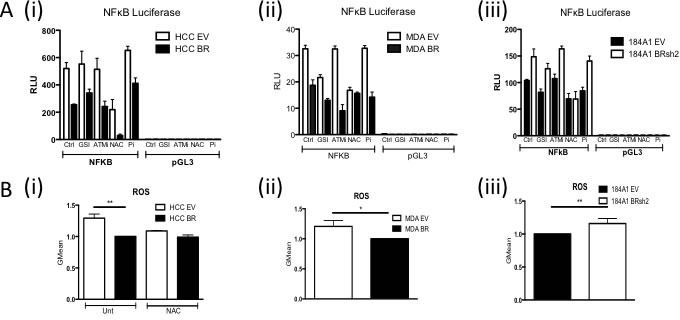
**A.** NFκB Luciferase Activity Assay of i) HCC EV and BR, (ii) MDA EV and BR and (iii) 184A1 EV and SH2 cells pre-treated for one hour with vehicle control (Ctrl), 250nM Gamma Secretase Inhibitor (GSI), 3.3μM ATM inhibitor (ATMi), 10 μM NAC or 10μM Parp inhibitor (Pi). Cells were then transfected with either NFkB reporter construct (NFκB) or the empty vector control (pGL3) with the relevant treatment. Renilla was used to normalise for transfection efficiency. Values are expressed as relative luciferase units (RLU) normalised to pGL3 and Renilla. **B.** Flow cytometry based analysis of Reactive Oxygen Species (ROS) using Carboxy-H2DCFDA in (i) HCC EV and BR cells treated with or without 10μM N-acetyl-L-cysteine (NAC), (ii) MDA EV and BR and (iii) 184A1 EV and SH2 cells.

In order to delineate how loss of BRCA1 function results in increased basal NFκB activity, we used a series of inhibitors to pathways known to be regulated in a BRCA1-dependent fashion that can impact on NFκB activity such as Notch [23], DNA Damage Response (ATM and Parp inhibitors) [24, 25] and Reactive Oxygen Species (ROS) [26] (Figure 2A). Inhibition of ROS using NAC consistently resulted in a loss of increased NFκB activity observed in the absence of functional BRCA1. Consistent with this observation, ROS levels were significantly higher in cells lacking functional BRCA1 compared to their BRCA1 proficient controls (Figure 2B).

**Figure 3:**
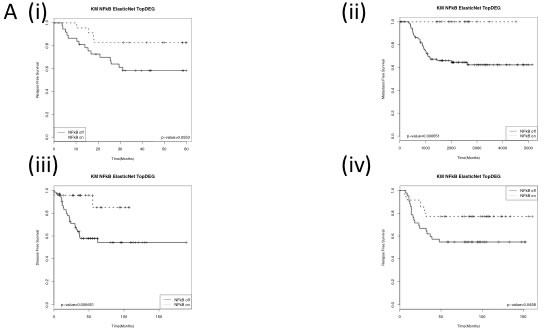
Kaplan Meier Curves of (i) the in- house Triple negative dataset and publically available (ii) GSE58812, (iii) GSE21653 and (iii) GSE2034 datasets stratified using the identified BRCA1-/NFκB+ (NFkB on) and non-BRCA1-/NFκB+ (NFkB off) groups Log-rank p-values are shown.

We next wanted to determine whether this observed biology was also present in breast cancer tumours. In order to achieve this, we created a cell line derived gene list of target genes specifically upregulated by NFκB in the absence of functional BRCA1. Microarray analysis was carried out on BRCA1 mutant and reconstituted cells (HCC EV and BR) cells with and without siRNA targeted against the p65 subunit of NFκB (Supp Figure 2A). This list was refined to the smallest gene list with the most robust and significant fold changes (Supp Figure 2B). This gene list was then used to interrogate a TNBC microarray data set enriched for BRCA1 mutations [27] in order to identify a molecular subgroup of breast cancers enriched for this biology and labelled as BRCA1-/NFκB+ (“NFκB on”) and all other tumours labelled as non-BRCA1-/NFκB+ (“NFκB off”). Unsupervised clustering was used to take into account that not all BRCA1 mutations result in the same dysfunction [10] and that not all BRCA1 wildtype tumours possess functional BRCA1 [4] (Supp Figure 2B(ii)). In order to develop a tumour derived classifier gene signature to identify the BRCA1-/NFκB+ subgroup, an ElasticNet computational analysis was applied (Supp Figure 2B(iii) and Supp Table 1) and further refined based on most statistically significant fold changes (Supp Figure 2B(iv) and Supp Table 2) comprising of 42 genes most of which (39/42g) are upregulated in the “NFκB on” subgroup. While this only contained one of our original cell line defined BRCA1/ NFκB target genes (CXCL10) this is not overly surprising as this is a tumour derived classifier. However, most of the cell line derived BRCA1/ NFκB genes were present in the differential gene list (DEG). Unfortunately, no clinical follow-up data was available for this cohort. Therefore, in order to determine the clinical significance of this “NFκB on” subgroup, the ElasticNet derived gene signature was applied to 4 additional TNBC datasets with available clinical follow up [18, 27-29]. Semi-supervised clustering, using the 42 gene signature, was used to identify the “NFκB on” and “NFκB off” subgroups (Supp. Figure 3) and relapse free survival analysed (Figure 3). As shown in the Kaplan Meier curves, the “NFκB on” subgroup has significantly better relapse free survival. Cox Proportional Hazard analysis (Table 1) shows the “NFκB on” subgroups were 2.5 - 5 times less likely to relapse. Similar results were also observed for overall survival where data was available (Supp Figure 4). Multivariate analysis on the in-house TNBC cohort showed that the NFκB signature was independent of age, tumour size, chemotherapy regime and lymphovascular invasion (LVI) status but not lymph node involvement (Supp Table 3). Consistent with our in-vitro data, the “NFκB on” subgroup also expressed higher levels of genes associated with high ROS levels compared to the “NFκB off” subgroup (Supp Figure 5).

**Figure 4:**
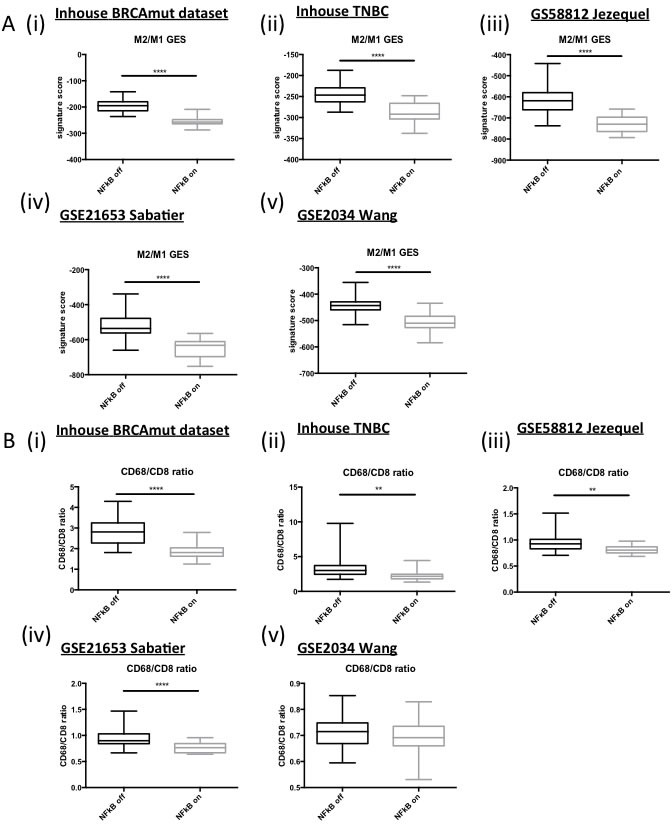
**A.** Box and Whisker plots of microarray derived M2/M1 Gene Expression Signature scores in (i) in house BRCA1 mutant dataset, (ii) in house TNBC dataset and publically available (iii) GSE58812, (iv) GSE21653 and (v) GSE2034 datasets. **B.** Box and Whisker plots of microarray derived CD68/CD8 expression ratios in (i) in house BRCA1 mutant dataset, (ii) in house TNBC dataset and publically available (iii) GSE58812, (iv) GSE21653 and (v) GSE2034 datasets.

Given that the majority of the genes within the ElasticNet derived genelist are involved in immune response and the previously discussed high levels of immune cell infiltrate observed in BRCA1-mutant and TNBC, the next obvious step was to investigate whether the tumour microenvironment of these tumours was different for the “NFκB on” *vs* “NFκB off” tumour sub groups. Therefore, a TMA matched to the in-house triple negative breast cancer cohort was utilised. Anti-CD8, -CD4 and -FOXP3 antibodies were used to assess cytotoxic, helper and regulatory T-cells, respectively. A separate score was given for T-cells located in the stroma *vs* those within the tumour nest (intratumour). No significant differences in stromal CD4, CD8 or both stromal and intratumoural FOXP3 were observed. However, there was a significant correlation of CD8 and CD4 positive T cells within the tumour nest and the “NFκB on” subgroup (Table 2 and Supp Figure 6). Numerous studies have shown that the polarisation state of the tumour-associated macrophages can strongly influence a tumour-promoting or -destroying microenvironment [30, 31]. Simply, M1-like macrophages promote a T_H_1 response and tumour destruction while the M2-like macrophages promote a T_H_2, CD8+ suppressive, tumour promoting response. Given the fact that a number of the genes up-regulated in the “NFκB on” subgroup are associated with interferon [32](Supp Figure 7) and the T_H_1 response (e.g. CXCL9, CXCL10), we postulated that the “NFκB on” subgroup may be associated with an M1-like macrophage environment. In order to test our hypothesis, we applied the M2/M1 gene expression signature (GES) developed by Jézéquel et al [18]. A highly significant correlation (p ≤ 0.0001) was observed between a low M2/M1 score (implying M1-like phenotype) and the “NFκB on” subgroup (Figure 4A). Alternatively, the CD68:CD8 ratio can also be analysed through microarray analysis and this has been shown to predict survival and chemotherapeutic response in breast cancer [33]. A high CD68:CD8 ratio implies a macrophage population suppressing the CD8 cytotoxic T-cells (e.g. M2-like) while a low score implies CD8+ T-cells mediated tumour suppression (e.g. M1-like). A significant correlation with a low CD68:CD8 ratio and the “NFκB on” subgroup was observed (Figure 4B). In order to validate the microarray-based gene expression data, the macrophage populations present in the TNBC TMA were assessed by IHC using the pan-macrophage marker, CD68. Consistent with the mRNA data, a higher CD68:CD8 ratio was observed in the “NFκB off” group compared to the “NFκB on” subgroup (Table 2 and Supp Figure 6). To assess M1/M2 polarisation, the CD14 and CD163 were also assessed by IHC [34, 35]. In keeping with the microarray-based data, higher CD14 staining was associated with “NFκB on” while higher CD163 was associated with the “NFκB off” subgroup (Table 2 and Supp Figure 6).

**Table 2:** *p*-values of Chi-Squared and/or Fisher's exact tests of IHC-based correlations between “NFkB on” or “NFkB off” subgroups and immune markers

		Chi Squared Test	Fisher's Exact Test
*N*= 53		*p*-value	
CD8	Intratumour	0.0133	0.0101
	Stroma	0.1124	-
CD4	Intratumour	0.0237	0.0817
	Stroma	0.4129	-
FOXP3	Intratumour	0.5061	-
	Stroma	0.4587	-
CD68	Intratumour	0.2120	-
	Stroma	0.3290	-
CD68:CD8		-	0.0612
CD14		0.0428	
CD163		0.0495	

**Figure 5:**
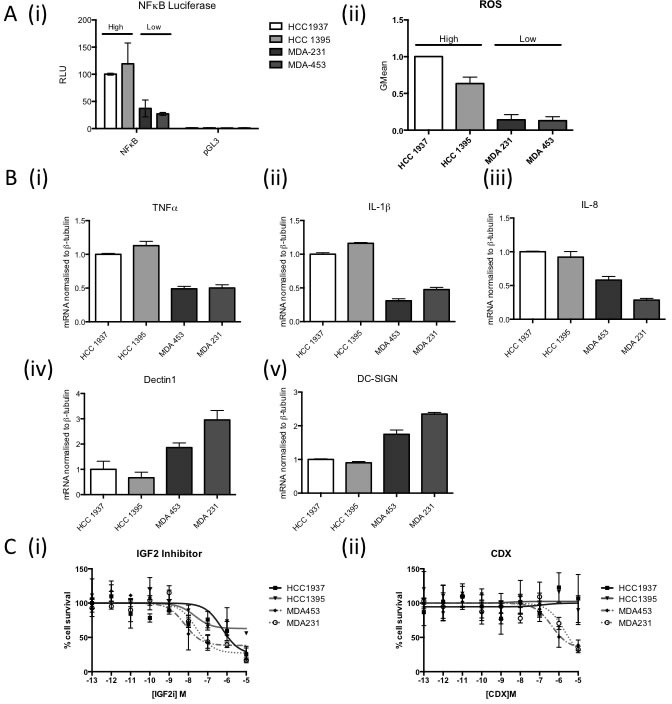
**A.** (i) NFκB Luciferase Activity Assay of 2 “NFκB-on” cell lines (HCC1937, HCC1395) and 2 “NFκB-off” cell lines (MDA231, MDA453). Cells were transfected with either NFkB reporter construct (NFκB) or the empty vector control (pGL3). Renilla was used to normalise for transfection efficiency. Values are expressed as relative luciferase units (RLU) normalised to pGL3 and Renilla. (ii) Flow cytometry based analysis of Reactive Oxygen Species (ROS) using Carboxy-H2DCFDA in the same cell lines as (i). **B.** Real time PCR analysis of M1 and M2 macrophage markers in THP-1 cells co-cultured with media from “NFkB on” (HCC1937, HCC1395) and “NFkB off” (MDA231, MDA453) cells for 24 hours. β-tubulin was used as a housekeeper. **C.** Dose response curve of “NFkB on” (HCC1937, HCC1395) and “NFkB off“(MDA231, MDA453) treated with (i) IGF2 inhibitor or (ii) Bicalutamide. Cells were treated with the indicated range of concentration of drug for 72hrs before cell viability was assessed by MTT. Cell survival was normalised to vehicle control (100%).

The results to date imply that the “NFκB on” subgroup respond well to the standard of care (SoC) DNA damaging chemotherapy used to treat TNBC (FEC +/− D). Therefore novel therapeutic strategies are required to improve outcome in the “NFκB off” subgroup. While isogenic cells lines are a crucial research tool to elucidate downstream events from modulation of a single genetic event, they can never fully recapitulate the changes that would be seen *in vivo* with selective pressure from the tumour microenvironment. Given the integral role of immune signalling in this study, we therefore wanted to identify representative cell lines that may more closely mimic the downstream consequences of loss of BRCA1 function and subsequent deregulation of basal NFκB *in vivo*. Using TNBC cell lines from within two publically available cell line datasets [36, 37] and semi-supervised clustering using the ElasticNet derived gene list, representative “NFκB on” (HCC1937 and HCC1395) and “NFκB off” (MDA231 and MDA453) cell lines were identified (Supp Figure 8). Interestingly, not all BRCA1 mutant cell lines were defined as “NFκB on”. This is in keeping with the assumption that not all BRCA1 mutations result in the same dysfunction [10] and our own data showing that not all BRCA1 mutant tumours are classified as “NFκB on” (Supp Figure 2). NFκB activity was shown to be higher in the “NFκB on” *vs* the “NFκB off” cell lines by luciferase activity assay (Figure 5A (i)) and consistent with the results in Figure 2, higher ROS levels were observed in the “NFκB on” compared to the “NFκB off” cell lines (Figure 5A (ii)). In order to demonstrate that the NFκB driven gene expression could regulate macrophage polarisation, tumour microenvironment and ultimately outcome in breast cancer cases, differentiated THP-1 cells were incubated with media from the four cell lines and then markers of M1 and M2 polarisation assessed by qPCR [38]. mRNA levels of the M1-associated markers (TNF-α, IL-1β and IL-8) were all significantly higher in the “NFκB on” compared to the “NFκB off” cell lines whilst the converse was seen with the M2-associated markers Dectin1 and DC-SIGN (Figure 5B). This implies that reprogramming of the microenvironment towards a more M1-like anti-tumour phenotype could be beneficial in the “NFκB off” subgroup. Furthermore, analysis of the ElasticNet derived genelist identified two “druggable” pathways up-regulated in the “NFκB off” compared to the “NFκB on” tumours namely the Androgen and IGF pathways. Microarray-based mRNA expression levels confirmed higher expression of IRS1, IGF2, IGF1R and AR in “NFκB off” compared to the “NFκB on” tumours in all five datasets (Supp Figure 9). In order to assess the therapeutic implication of this, inhibitors against IGF2 and androgen receptor were tested in the four representative cell lines. “NFκB off” cell lines were significantly more sensitive to inhibition of these pathways than the “NFκB on” cell lines (Figure 5C).

## DISCUSSION

In this study, we have shown that the absence of functional BRCA1, basal NFκB activity is increased due to increased ROS *in vitro*. This biology is found in a subset of BRCA1 mutant and TNBC cases and confers good outcome. The increased NFκB signalling results in an anti-tumour microenvironment which may allow CD8+ cytotoxic T cells to suppress tumour progression. However, tumours lacking this NFκB-driven biology have a more tumour-promoting environment and so are associated with poorer prognosis when treated with chemotherapy. Tumour-derived gene expression data and cell line models imply that these tumours may benefit from alternative treatment strategies such as reprogramming the microenvironment and targeting the IGF and AR signalling pathways.

This work highlights the fact that BRCA1-mutant and TNBCs are a heterogeneous groups of cancers that are not benefitting from the current “one size fits all” standard of care chemotherapy. This is further exemplified by use of PARP inhibitors to exploit the DNA repair defect of BRCA1 and BRCA2 mutant breast cancers. Despite good response rates in early clinical trials, no survival benefit has been demonstrated in BRCA1 mutant or TNBCs [8]. Therefore, biomarkers and potential therapeutic strategies must be developed around discreet biology downstream of specific modes of BRCA1-dysfunction. Indeed, a study by Fernandez-Ramies et al demonstrated that the immune response signature associated with ER-negative BRCA1 mutant tumours was modified by the type of BRCA1 mutation. BRCA1 mutant tumours harbouring truncating mutations (that probably led to a complete absence of protein through nonsense-mediated decay (NMD)) had a lower magnitude of expression of the immune genes compared to those harbouring missense mutations (resulting in an aberrant but still present BRCA1 protein) and this was underpinned by differing expression levels of the NFκB transcription factors [20]. Consistent with this, the majority of the tumours associated with that “NFκB on” subgroup harbour C-terminal mutations, which tend to avoid NMD and are expressed at relatively normal levels [39]. Furthermore, these mutations are commonly associated with loss of transcription regulation by BRCA1 [40] which is in keeping with our observations and known BRCA1-dependent regulation of NFκB function [21].

Our current study highlights the known variation in outcome of patients with TNBC where some patients do very poorly, relapse and die within the first 3 years while patients who do not recur within this period tend to have a much better prognosis [41]. Our data would suggest that the “NFκB on” subgroup respond well to the current standard of care DNA damaging chemotherapy (FEC +/−D) due in part to their favourable microenvironment. We would suggest however, that these patients probably would not benefit from the addition of docetaxel to their regime regardless of lymph-node involvement, given that tumours with dysfunctional BRCA1 are less likely to respond to anti-microtubule agents [42]. Counter-intuitively, it is interesting to note higher gene expression of the PD1 ligand, PD-L1 (CD274) in this subgroup. This implies that these tumours may also benefit from an immune checkpoint blockade strategy. This emphasises the complexity of dialogue between the tumour cell and the cells within the microenvironment such as T-cells and macrophages [31]. We suggest that the NFκB-driven signal from within the tumour cells promotes a M1/T_H_1 microenvironment that then produce their own signals to enhance and maintain this and influence responses to chemotherapy and overall outcome. Therefore, the “NFκB off” subgroup may benefit from a more taxane-based chemotherapeutic regime (FEC-D rather than FEC as first line treatment) or targeted therapies (alone or in combination) to re-program the microenvironment towards a more M1-like anti-tumour scenario. A number of these drugs have been developed (e.g. the CD-20 targeting agent, Rituximab) and show promise in *in vivo* models and in patients [30]. Furthermore, reprogramming of macrophages may be a more favourable approach compared to eradication, as they may be required for interaction with other components of the microenvironment [43]. In addition, our data suggests that use of therapies targeting the IGF and AR signalling pathway may also benefit this subgroup. These pathways have already been identified as tractable drug targets within TNBC with clinical trial results showing promise [44]. We believe that this NFκB-driven biology may allow stratification to predict who is likely to respond from such therapies.

In summary, we have identified a BRCA1-deficient, NFκB-driven biology that predicts good outcome in TNBC due to the promotion of a favourable tumour microenvironment where immune targeting of the tumours is more efficient. Knowledge of this can be used to improve poorer outcome patients through macrophage reprogramming or use of specific targeted therapies.

## MATERIALS AND METHODS

### Cell lines

All cell lines were purchased from ATCC and maintained as directed except for the 184A1 cells which were a kind gift from Dr Martha Stampfer (University of California) and maintained as previously described [45]. Cell lines were characterised by isoenzyme/cytochrome c oxidase I (COI) assay and short tandem repeat (STR) analysis by the cell bank. Full details of the HCC-EV/BR and MDA468-EV/BR cell lines are provided in [46]. 184A1-EV and -BRsh2 cells were generated by stable lentivirus transfection of the 184A1 cells with pll3.7-EV or BRsh2 respectively (a kind gift from Prof. Wicha). Infected cells were selected in the presence of 1μg/μl puromycin. For drug treatments, cells were treated with the relevant concentration of IGF2 inhibitor (Sigma, UK) or Bicalutamide (Sigma, UK) for 72hrs before cell viability was assessed by MTT (Sigma, UK).

### Short interfering RNA (siRNA)

Transfections were done using RNAiMax reagent (Invitrogen, UK), as outlined in the manufacturer's instructions. siRNA oligonucleotide were obtained from Eurofins and used at a final concentration of 10nM.

### Western blot analysis

Protein lysates were extracted in EDTA Lysis Buffer (ELB) (0.25M NaCl, 0.1% IEPGAL, 0.25M Hepes, 5mM EDTA, 0.5mM DTT), separated on a SDS PAGE gel, transferred to a PVDF membrane followed by immunoblotting. Antibodies are previously described in [23]

### RNA extraction, reverse transcription and real time quantitative PCR (RqPCR)

RNA was extracted using RNA STAT60 Total RNA extraction Reagent (Tel-Test Inc, USA), reverse transcribed using the Transcriptor First Strand cDNA Synthesis kit (Roche, UK) and RqPCR analysis performed on the LC96 (Roche, UK) using Sybr Green (Roche, UK) according to the manufacturer's instructions. Primers used are listed in Supplementary Data and [38].

### Luciferase assays

NFκB-pGL3 has been previously described [47]. Cells were co-transfected with the relevant Luciferase constructs and Renilla using GeneJuice (Novagen, Germany) according to the manufacturer's instructions. After 24hrs, cells were lysed with Passive Lysis Buffer (Promega, UK) and Luciferase and Renilla activity assessed by luminescence using D-Luciferin and Coelenterazine as substrates, respectively. For treatments with Gamma Secretase Inhibitor (Calbiochem, Germany), ATM inhibitor (KU60019 Tocris Biosciences, UK), NAC (Sigma, UK) and Parp Inhibitor (Olaparib, Selleckchem, UK), cells were pre-treated for 1hr prior to transfections with luciferase constructs and maintained in drug for the length of the experiment.

### Reactive oxygen species detection

Cells were incubated with 5μM CM-H2DCF-DA (Invitrogen, UK) for 30 minutes followed by flow cytometry. TeBOOH was included as a positive control in all experiments.

### Tissue microarrays (TMAs)

The breast cancer TMAs used in this study were constructed from Formalin-fixed paraffin-embedded primary tumour blocks by the Northern Ireland Biobank and have been previously described [48]. Each tumour sample was represented by three independent cores. TNBC cases from within this cohort were identified from associated clinical and pathological information. Repeat IHC for ER, PR and HER2 on the TMA sections confirmed the TNBC status of this case cohort. Full details of antibodies used are listed in Supplementary Data. All antibodies were scored independently by two histopathologists blinded to patient clinicopathological and outcome data. Immune markers were scored on a 0-3 scoring system with representative images in Supplementary Figure 10.

### Microarrays

Microarray analyses were performed as previously described on the Almac Breast DSA [49]. NFκB target genes were determined by identifying BRCA1 and p65 regulated genes independently (using the R package “limma”) and then overlapping the gene lists. TNBC samples were identified (if required) from within the public datasets [18, 27-29] using the associated clinical information. BRCA2 mutant samples were excluded from discovery dataset [27].

### ElasticNet

The ElasticNet regularization procedure was performed using the R package “glmnet” [50]. The optimal lambda was chosen based on a 10-fold cross-validation. The ElasticNet regularization is a convex combination of the ridge and the lasso penalty with a weighting parameter “alpha” (0.3 was used). Bootstrapping (x100) followed by a hypergeometric test to identify non-random features was used for feature selection.

### Survival analysis

All Kaplan Meier Curves and Hazard Ratio Calculations were carried out using the R package “Survival”.

### Statistical analysis

All relevant data was analyzed by two-tailed Students *t*-test. All data was deemed significant with a p-value of at least < 0.05. All *p*-values are included in Supplementary Data.

## SUPPLEMENTARY MATERIAL FIGURES AND TABLES












