# Supplementary material for: A BRCA1 deficient, NFκB driven immune signal predicts good outcome in triple negative breast cancer
Source: Oncotarget. 2016 Mar 2;7(15):19884–96. doi: 10.18632/oncotarget.7865 (PMC4991425; doi:10.18632/oncotarget.7865)
Supplement: Supplementary file 1 [file oncotarget-07-19884-s001.pdf]

# A BRCA1 deficient, NFκB driven immune signal predicts good outcome in triple negative breast cancer

**Supplementary Material**

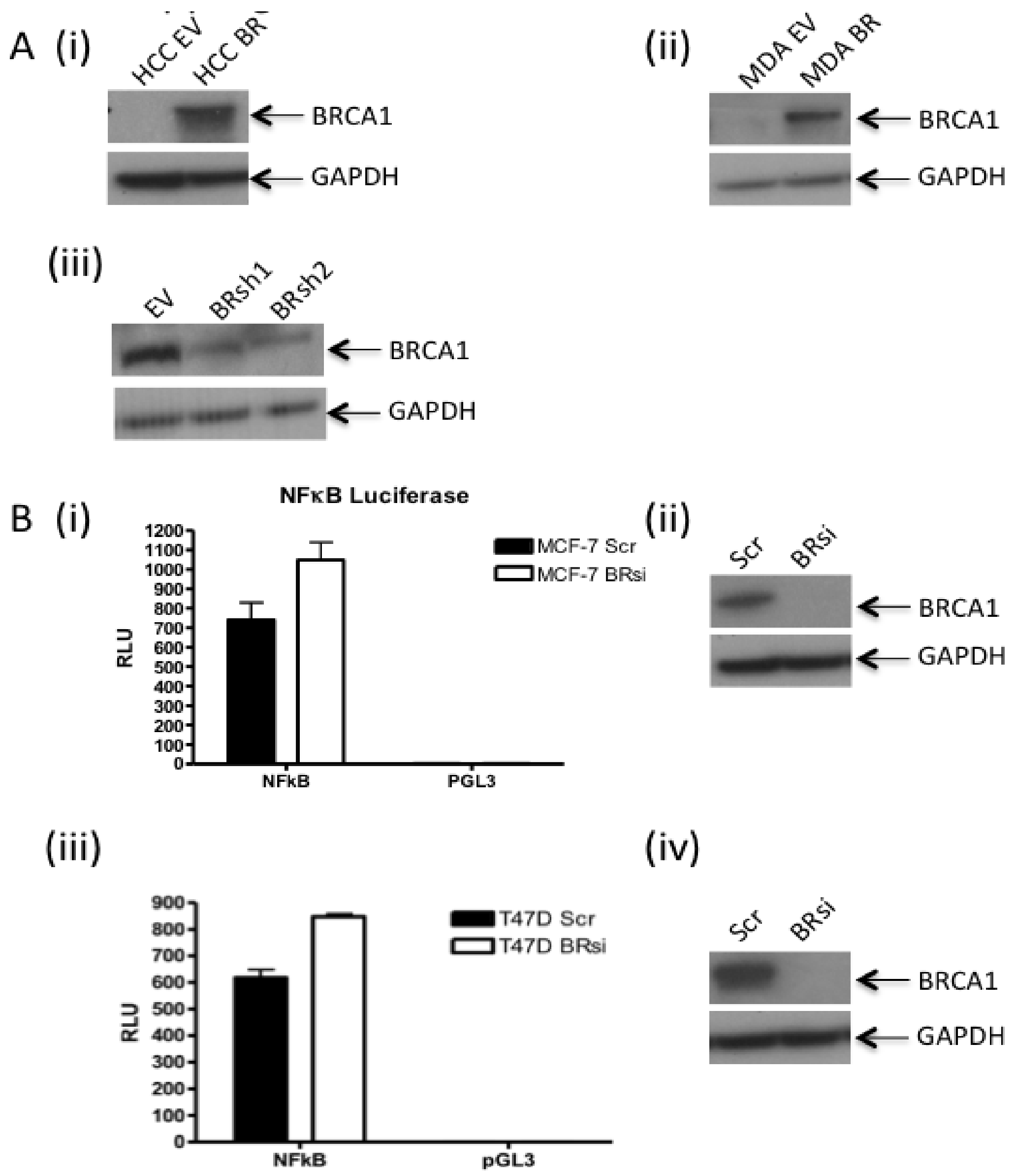


Supp Figure 1

**(A)** Western blot of (i) HCC1937 and (ii) MDA 468 cells stably transfected with empty vector (EV) or wildtype BRCA1 (BR) or (iii) 184A1 cells stably transfected with a BRCA1 shRNA (BRsh2) or empty vector control (EV). Blots were then

probed with BRCA1 or GAPDH as a loading control. **(B)** NFκB Luciferase Activity Assay of (i) MCF-7, (iii) T47D cells transiently transfected with scrambled (scr) or BRCA1 specific (BRsi) siRNA. Cells were transfected with either NFkB reporter construct (NFκB) or the empty vector control (pGL3). Renilla was used to normalise for transfection efficiency. Values are expressed as relative luciferase units (RLU) normalised to pGL3 and Renilla. Western blot of the same cells were probed for BRCA1 and GAPDH as a loading control (ii) and (iv).

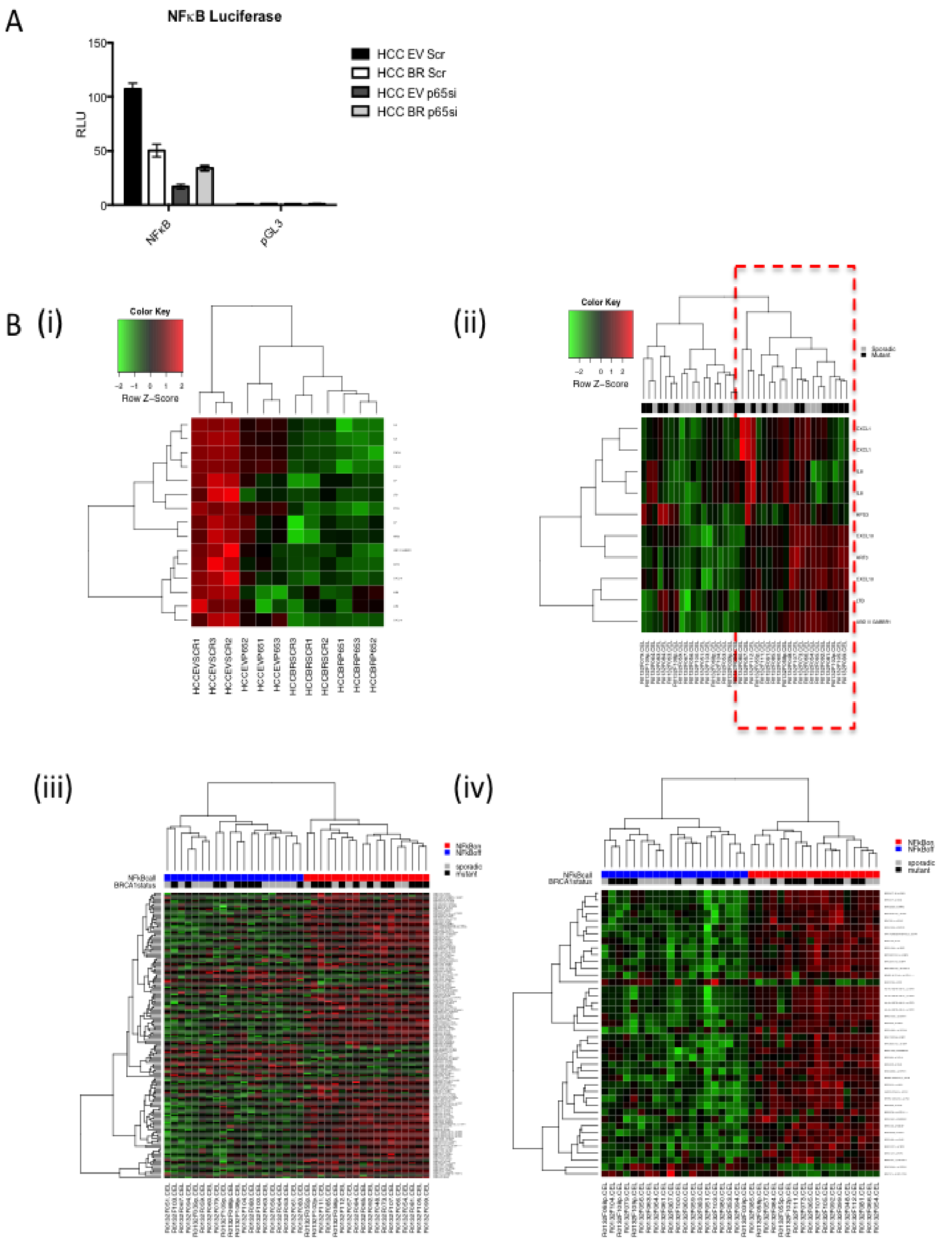


Supp Figure 2

(A) NFκB Luciferase Activity Assay of HCC1937 cells stably transfected with either empty vector control (EV) or wildtype BRCA1 (BR) followed by transient transfected with scrambled (scr) or p65 specific (p65si) siRNA. Cells were transfected with either NFkB reporter construct (NFκB) or the empty vector control (pGL3). Renilla

was used to normalise for transfection efficiency. Values are expressed as relative luciferase units (RLU) normalised to pGL3 and Renilla. (B)(i) Hierarchical semi-supervised analysis with the top 15 differentially regulated genes between the same samples as (A). (ii) Hierarchical semi-supervised analysis with the top 15 differentially regulated genes identified in (i) using the BRCA1 mutant cohort samples. Red boxes define the tumours determined to be BRCA1-/NFκB+. Hierarchical semi-supervised analysis with the full (iii) and (iv) refined ElasticNet derived gene list using the same samples as (ii).

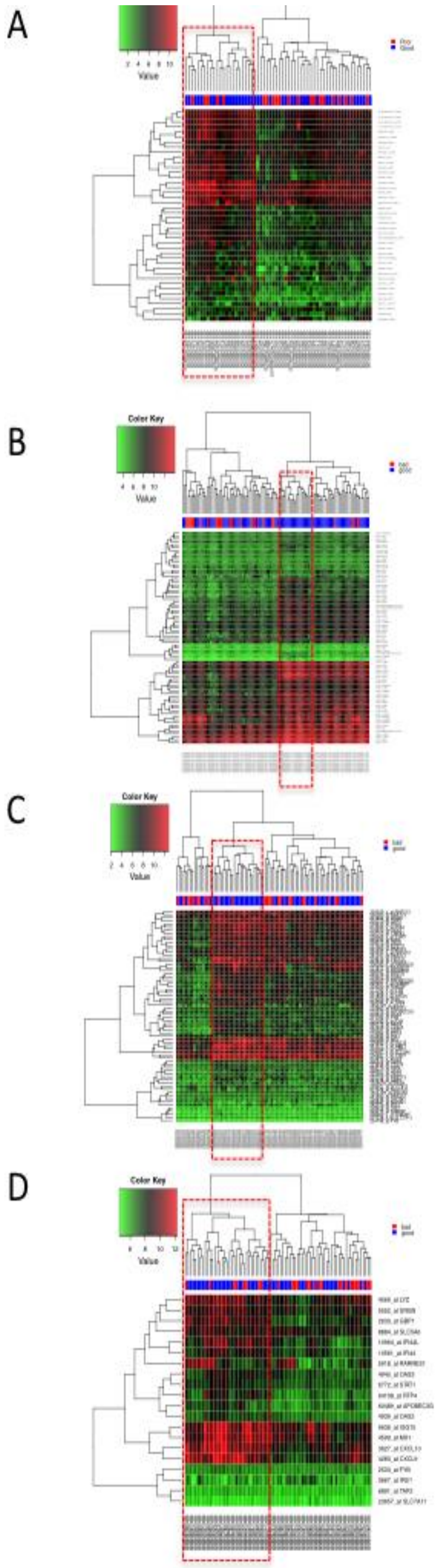
A
Value
B
Color Key
Value
C
Color Key
Value
D
Color Key
Value

Supp Figure 3

Hierarchical semi-supervised analysis with using the elastic-net derived gene list of **(A)**(i) in house Triple negative dataset and publically available **(B)**(i) GSE58812, **(C)(**i) GSE21653 and **(D)** (i) GSE2034 datasets. Red boxes define the tumours determined to be BRCA1-/NFκB+.

A

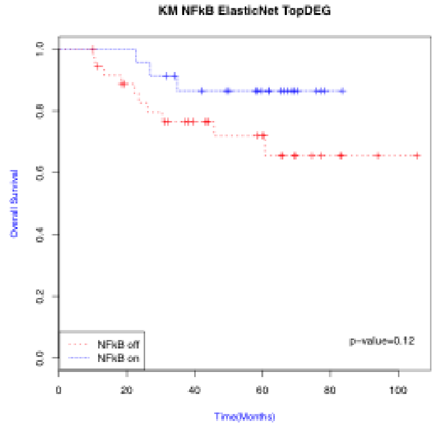


B

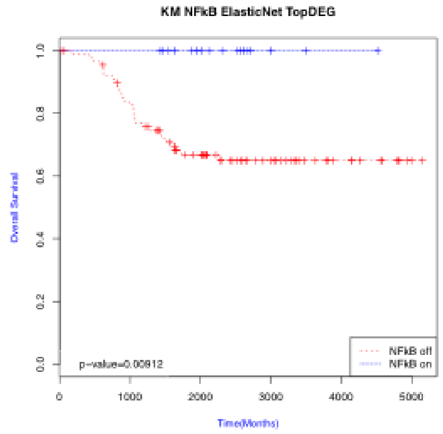


Supp Figure 4

Kaplan Meier Curves of overall survival of **(A)** the in- house Triple negative or **(B)** publically available GSE58812 datasets stratified using the identified BRCA1-/NFκB+ (NFkB on) and non-BRCA1-/NFκB+ (NFkB off) groups. Log-rank p-values are shown.

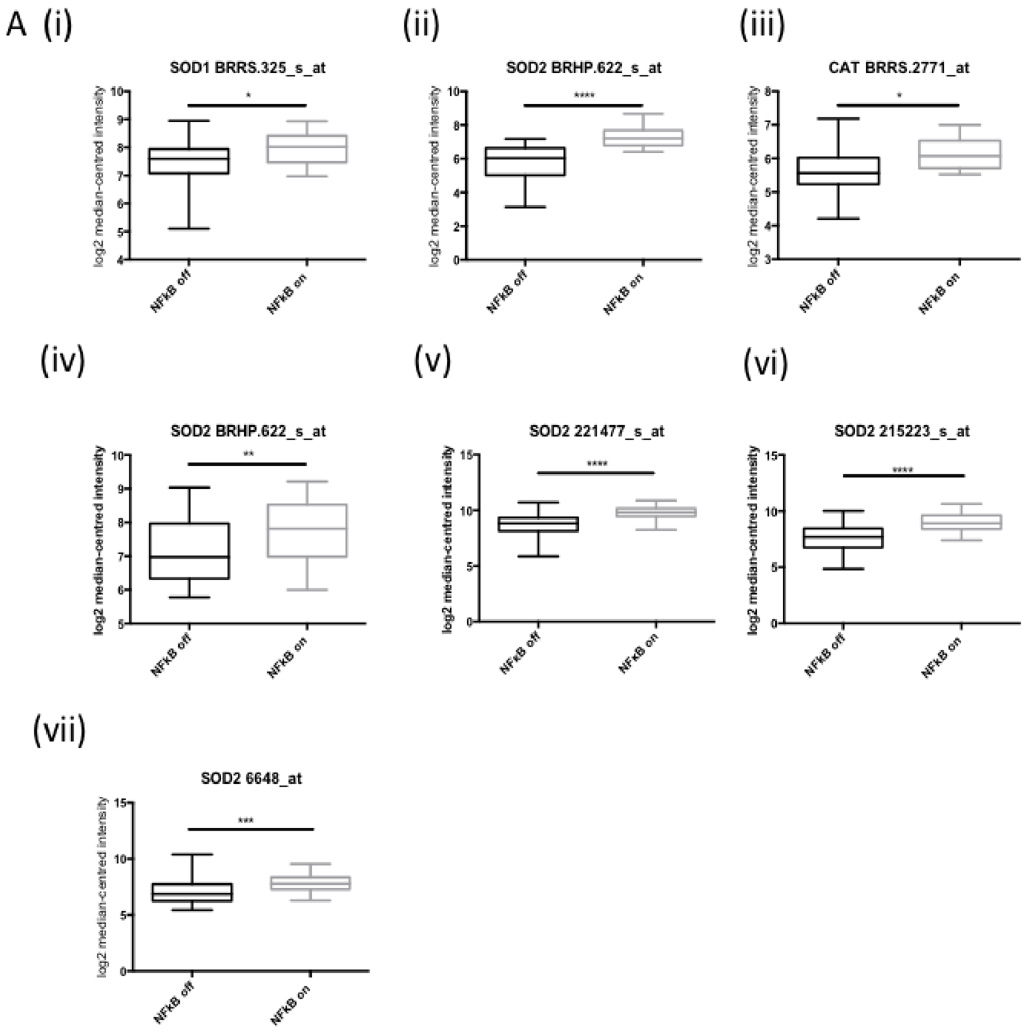


Supp Figure 5

Box and Whisker plots of microarray derived expression levels of ROS related genes in **(A)**(i-iii) BRCA1 mutant, (iv), the in-house triple negative or the publically available (v) GSE58812, (vi) GSE21653 or (vii) GSE2034 data sets.

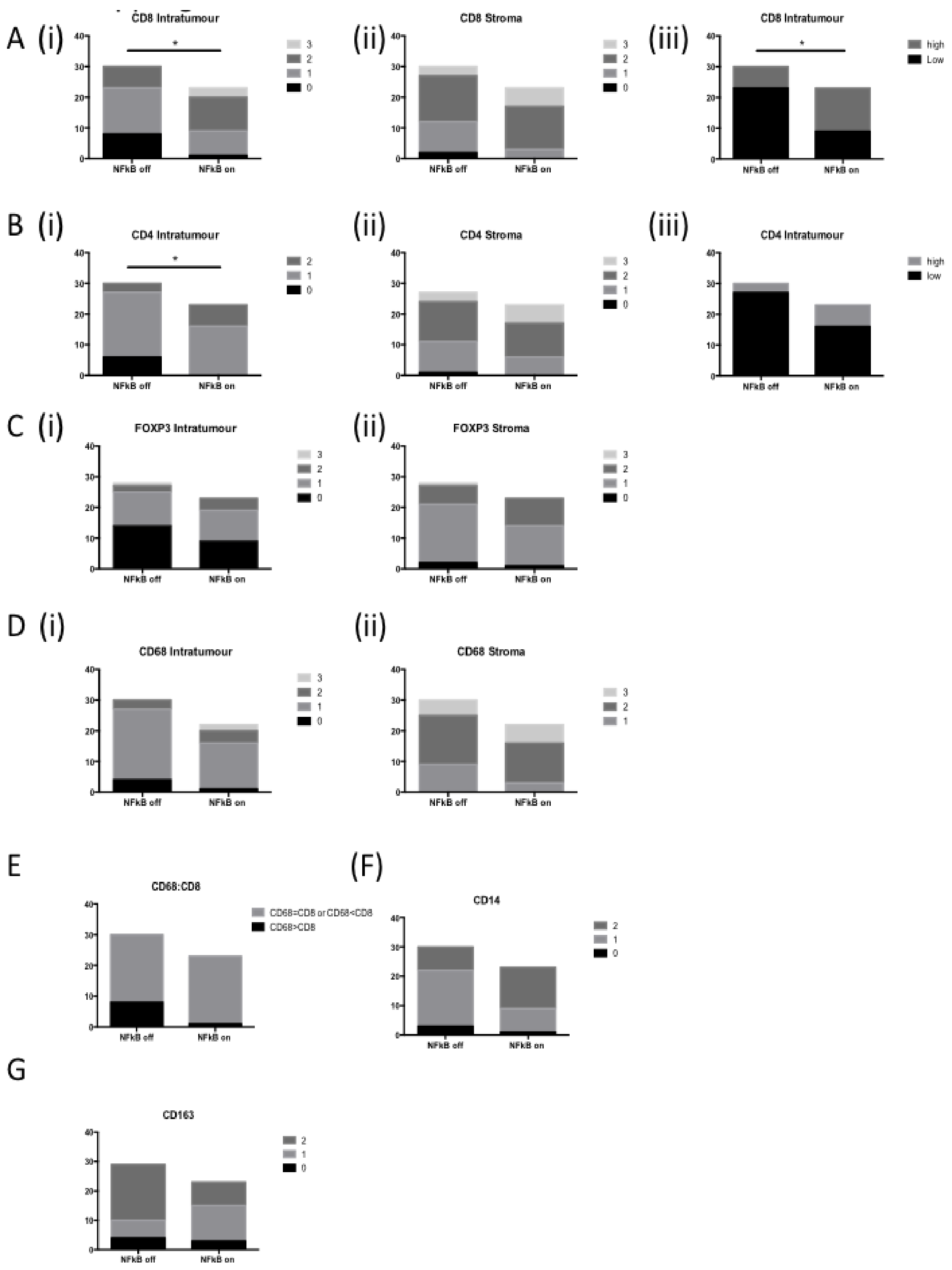


Supp Figure 6

Stacked bar graphs of IHC scores for **(A)** CD8, **(B)** CD4, **(C)** FOXP3, **(D)** CD68, **(E)** CD68:CD8 ratio, **(F)** CD14 and **(G)** CD163.

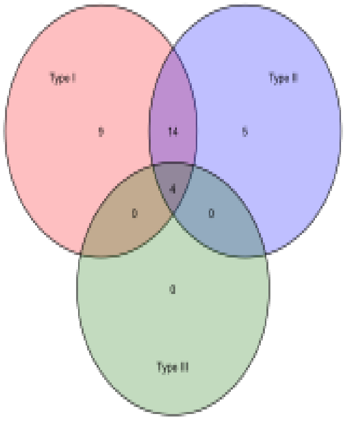


Supp Figure 7

Venn Diagram of refined ElasticNet genes classified according to Interferon Class using the online tool INTERFEROME v2.0.

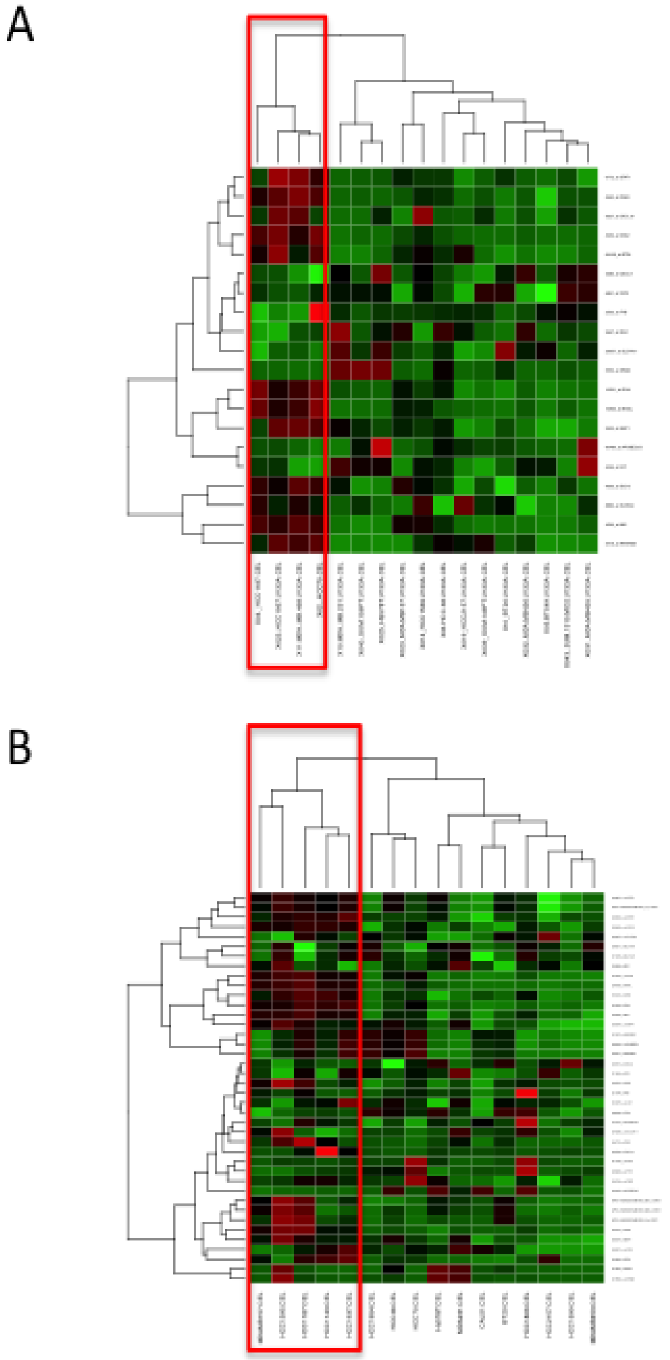


Supp Figure 8

Hierarchical semi-supervised analysis with the refined ElasticNet derived gene list using the publically available cell line datasets **(A)** E-TABM-157 and **(B)** E-MTAB-783. Red boxes define the cell lines determined to be BRCA1-/NFκB+.

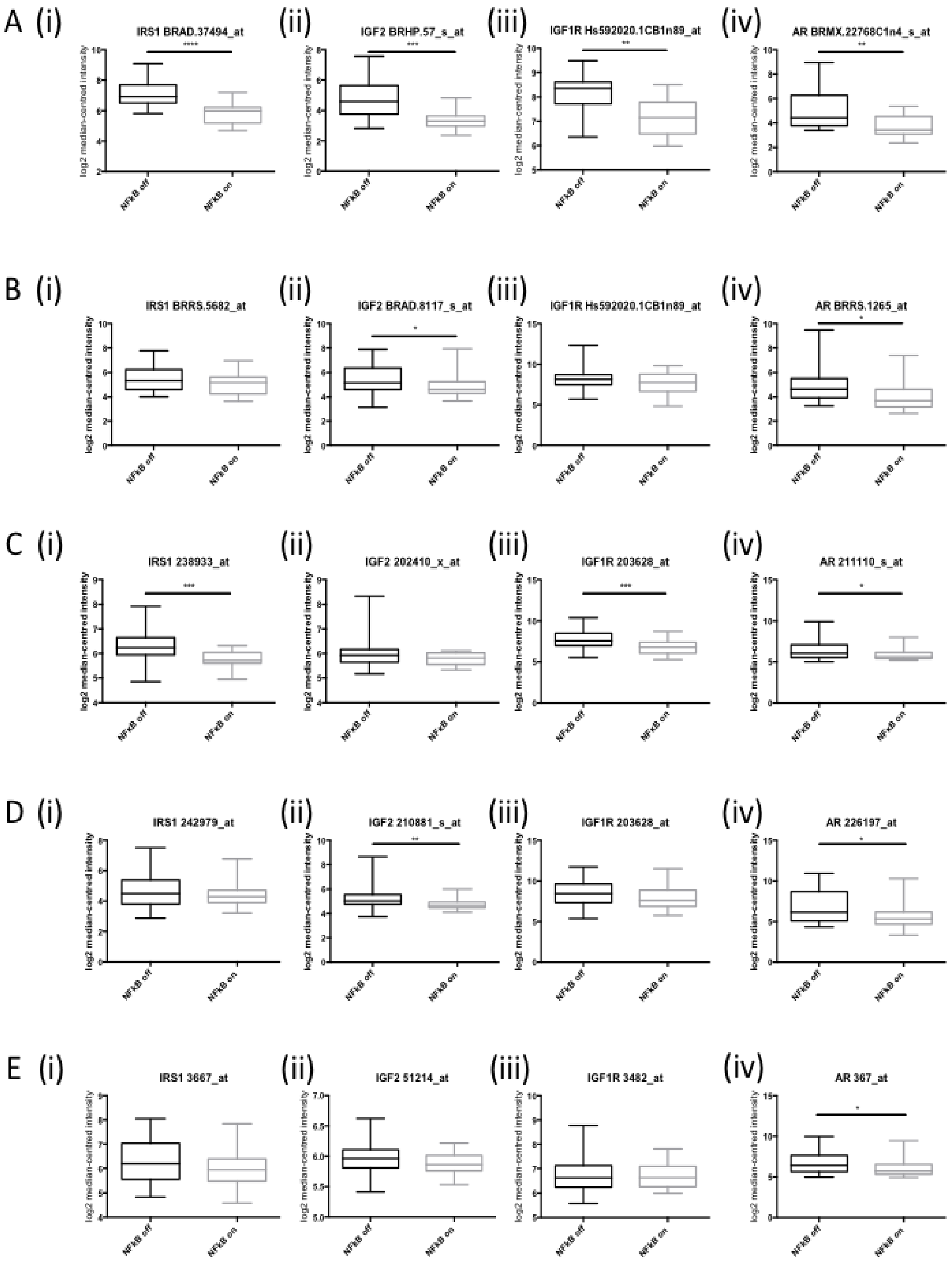


Supp Figure 9

Box and Whisker plots of microarray derived expression levels of IGF and AR related genes in **(A)**BRCA1 mutant, **(B)** the in-house triple negative or the publically available **(C)** GSE58812, **(D)** GSE21653 or **(E)** GSE2034 data sets.

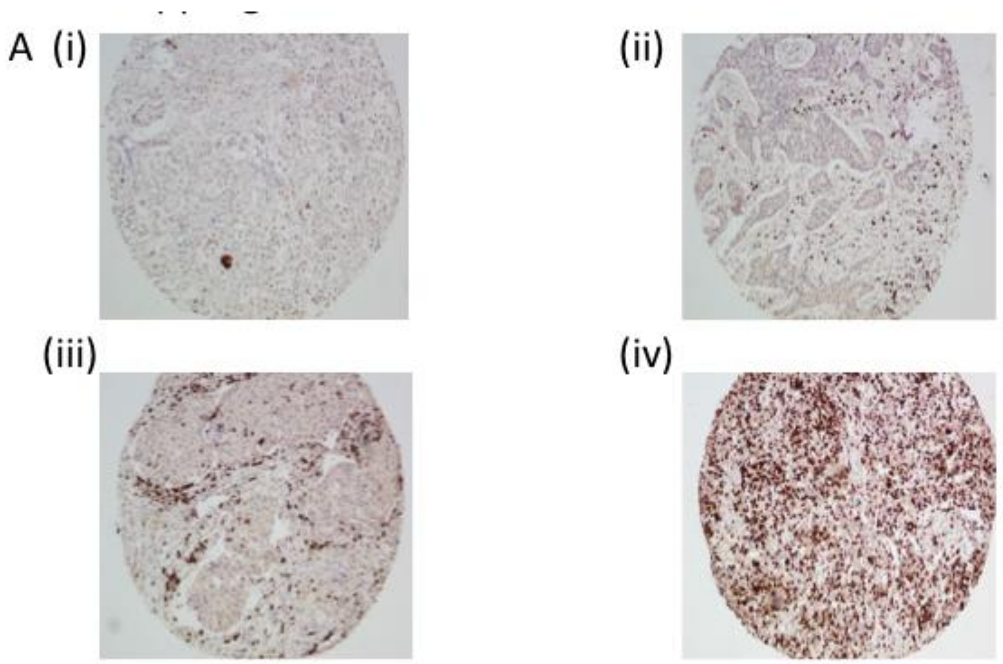


Supp Figure 10

Representative images of IHC scoring system for immune markers.  Where necessary, 2 scores were assigned – intratumoural and stromal.  Images representing (i) 0/0, (ii) 0/2, (iii) 1/3 and (iv) 3/3 are shown.

Supp Table 1:

Full ElasticNet genelist.

Supp Table 2:

Refined ElasticNet gene list.

Supp Table 3:

Multivariate Cox Proportional Hazard Ratio analysis of relapse free survival in the in-house triple negative dataset.
