# Supplementary material for: A BRCA1 deficient, NFκB driven immune signal predicts good outcome in triple negative breast cancer
Source: Oncotarget. 2016 Mar 2;7(15):19884–96. doi: 10.18632/oncotarget.7865 (PMC4991425; doi:10.18632/oncotarget.7865)
Supplement: Supplementary file 4 [file oncotarget-07-19884-s004.pdf]

| Relapse Free Survival | | | Cox PH - Multivariate | | |
|---|---|---|---|---|---|
| | | N (n) 60 (19) | HR | % 95 CI | p-value |
| BRCA1/NFkB | off | 37 (15) | 1 | | |
| | on | 23 (4) | 0.32598 | 0.098-1.105 | 0.0721 |
| Age | <40 | 8 (3) | 1 | | |
| | 40/49 | 23 (6) | 1.04 | 0.227-4.768 | 0.958 |
| | 50/59 | 12 (5) | 0.628 | 0.14-2.806 | 0.5432 |
| | 60+ | 17 (5) | 0.444 | 0.09-2.115 | 0.3084 |
| T Code | 1 | 20 (6) | 1 | | |
| | 2-4 | 40 (13) | 0.518 | 0.165-1.631 | 0.2616 |
| N code | 0 | 39 (7) | 1 | | |
| | 1-3 | 21 (12) | 3.93 | 0.99-15.622 | 0.0515 |
| Chemo | FEC | 52 (15) | 1 | | |
| | FEC-D | 8 (4) | 0.717 | 0.18-2.816 | 0.6338 |
| LVI present | no | 31 (4) | 1 | | |
| | yes | 29 (15) | 3.2 | 0.724-14.19 | 0.124 |

Table2
Multivariate Cox Proportional Hazard Ratio analysis of relapse free survival in the in-house triple negative dataset.
